# Coeliac Disease and Mast Cells

**DOI:** 10.3390/ijms20143400

**Published:** 2019-07-11

**Authors:** Barbara Frossi, Marco De Carli, Antonino Calabrò

**Affiliations:** 1 Department of Medical and Biological Science, University of Udine, 33100 Udine, Italy; 2 Second Unit of Internal Medicine, University Hospital of Udine, 33100 Udine, Italy; 3 Department of Experimental and Clinical Biomedical Sciences “Mario Serio”, University Hospital of Florence, 50134 Florence, Italy; 4 Tuscany Referral Center for Adult Coeliac Disease, AOU Careggi, 50134 Florence, Italy

**Keywords:** mast cells, coeliac disease, gliadin immunology, p31-43 fragment, 33-mer peptide, 25-mer fragment

## Abstract

Over the last decades, there has been an impressive progress in our understanding of coeliac disease pathogenesis and it has become clear that the disorder is the final result of complex interactions of environmental, genetic, and immunological factors. Coeliac disease is now considered a prototype of T-cell-mediated disease characterized by loss of tolerance to dietary gluten and the targeted killing of enterocytes by T-cell receptor αβ intraepithelial lymphocytes. Accumulating evidence, however, indicates that the induction of a gluten-specific T helper-1 response must be preceded by the activation of the innate immune system. Mast cells are key players of the innate immune response and contribute to the pathogenesis of a multitude of diseases. Here, we review the results of studies aimed at investigating the role of mast cells in the pathogenesis of coeliac disease, showing that these cells increase in number during the progression of the disease and contribute to define a pro-inflammatory microenvironment.

## 1. Introduction

Coeliac disease (CD) is a human autoimmune-like disorder characterized by chronic inflammation of the small intestine induced by the ingestion of proline- and glutamine-rich wheat gluten and other gluten-related proteins from rye and barley. Different symptoms and associated conditions can be the hallmark of the disease [1]. The prevalence of the disease is approximately 1% in the western world, although the majority of individuals have not been diagnosed [2]. The T-cell mediated damage of the intestinal mucosa that causes malabsorption is histologically represented by villous atrophy, crypt hyperplasia, and infiltration of lymphoid cells both in the epithelium and in the *lamina propria*. Many patients have less overt intestinal changes and malabsorption may be limited to micronutrients. CD is presently classified as symptomatic disease—which includes gastrointestinal and extraintestinal manifestations–, and subclinical disease, which includes those subjects that do not have symptoms and signs of overt clinical suspicion for the disease. Symptomatic CD can be further classified into classical and atypical forms. Classical forms are those characterized by the typical picture of malabsorption (i.e. diarrhoea and/or weight loss) whereas the term atypical refers to patients with minimal, transient, or apparently unrelated symptoms. In recent years, the pattern of clinical presentation of adult CD has changed and atypical presentation is now the most prevalent form of coeliac disease [1]. In addition, CD subjects have an increased prevalence of autoimmune diseases, which may be found in 35% of patients [3].

A definite diagnosis of CD is based on histological changes, including intraepithelial lymphocytosis, crypt hyperplasia, and varying degrees of villous atrophy, graded according to a classification system proposed by Marsh and modified by Oberhuber (Marsh I-IIIc) [4].

The pathogenesis of the tissue damage in CD is related to genetic and environment factors and is the consequence of complex mechanisms of activation belonged to both the acquired and the innate branch of the immune system. Among innate immune cells, mast cells (MCs) were previously associated with CD, but their role in the pathogenesis of the disease has been for long time unknown.

In this review we will briefly describe the main features of the immune response against gluten, genetics as well as innate and acquired immune reactions. Then we will focus on the role of MC in the complex scenario of the CD by: (i) describing the contribution of the MCs and of MC mediators in the control of the intestinal functions; (ii) reviewing the past literature concerning the role of MCs in the pathogenesis of coeliac disease; (iii) by presenting the most recent data that demonstrate the active role of MC in the onset and progression of the CD. The aim of the review is to highlight the MC as a crucial player in the CD.

## 2. Genetic and Immune Features of Coeliac Disease

CD has a strong genetic association, mainly with the genes coding for HLA-DQ2 and, to a lesser extent, with the genes coding for HLA-DQ8. Gluten-reactive CD4+ T-cells have been isolated from the small intestine of CD patients (but not from controls). These cells predominantly recognize peptides derived by gluten in which glutamine residues at certain positions have been converted to glutamate by the tissue transglutaminase 2 (TG2). This is the same enzyme that is the target of disease-specific serum autoantibodies, which are detected in untreated CD patients [5].

Gluten peptides, once deamidated in situ by TG2, activate a T-cell specific response, which is believed to play an essential role in the pathogenesis of CD. The anti-gluten immune response in CD is further characterized by increased epithelial permeability [6] and increased influx of gluten peptides to the *lamina propria* [7].

The genetic predisposition may favour the onset of CD from early childhood to old age. Although HLA-DQ2 and HLA-DQ8 haplotypes predispose to CD, only a fraction of the carriers develops the disease. The role of adaptive immunity in the pathogenesis of CD has been extensively studied. Many different gluten peptides derived from gliadins and glutenins are able to stimulate CD4+ T-cells of the small intestinal mucosa of CD patients [8,9]. Deamidation of these peptides by TG2 increases their affinity for HLA-DQ2 [10,11] and results in strong activation of T-cell response dominated by IFN-γ production [12,13] and additionally by IL-4, IL-5, IL-10, TNF and TGF-β [12]. Gluten-specific T-cells also produce IL-21, but not IL-22 or IL-17 [13]. However, it has been reported that mucosal IL-17 is elevated in CD in later stages of mucosal inflammation, when villous atrophy has developed [14].

In wheat, gluten can be subdivided into gliadin and glutenin components. The gliadin proteins are classified in α/β-, γ- and ω-gliadins, whereas glutenin are classified into high (MW = 67,000–88,000) and low molecular weight subunits (MW = 32,000–35,000).

**Figure 1:**
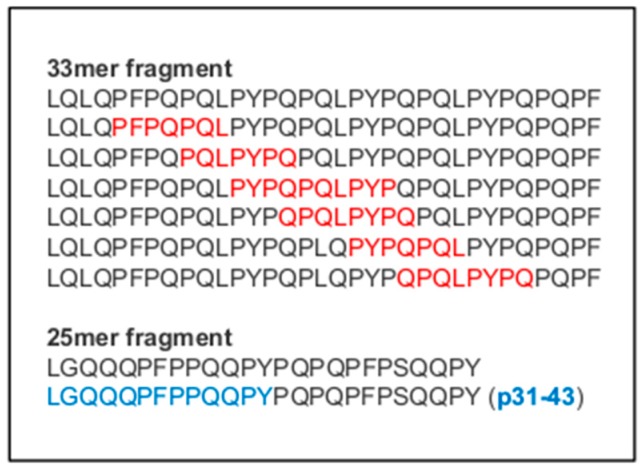
Amino acid sequence of the 33-mer and 25-mer fragments of the α-gliadin. Major immunogenic epitopes are indicated in red, while the sequence of the non-immunogenic fragment p31-43 is shown in blue.

Fifty different T-cell specific gluten epitopes both in native and deamidated forms has been described [15], but the major gluten protein involved in CD is the α-gliadin isoform containing the 33-mer peptide (Figure 1), which is considered the most immunogenic fragment. The 33-mer peptide is produced by digestion of α-gliadin proteins and it is resistant to enzymatic activity of gastric, pancreatic and intestinal brush-border membrane enzymes [15]. The 33-mer fragment contains six overlapping copies of three different HLA-DQ2-restricetd T-cell epitopes. Interestingly, this peptide can activate T-cell by binding to HLA-DQ2 antigens directly on the surface of the antigen presenting cells without needing further intracellular processing [16]. Therefore, gliadin can bypass the conventional intracellular processing by undergoing extensive luminal and brush-border proteolysis and the resultant antigenic peptides can be subjected to direct extracellular binding to surface HLA-DQ2.

The role of other cell types has also been evaluated. CD8+ T-cells are particularly abundant in the coeliac intestinal mucosa and the massive infiltration particularly of the epithelium by these cells is one of the diagnostic hallmark of CD and is present even in mild forms of the disease [17]. These cells express activating natural killer receptors (NKRs) that recognize stress induced ligands and they can destroy distressed epithelial cells independently from their TCR specificity [18]. Gluten-reactive CD8+ T-cells can be isolated from intestinal biopsies of both treated and untreated CD patients [19]. However, these gluten-specific CD8+ T-cells in the coeliac mucosa are located in the *lamina propria* and not in the epithelium where tissue destruction is detected [20]. Therefore, the role of gluten-specific CD8+ T-cell response is still to be fully elucidated.

Although the role of adaptive immunity is well established, little is known about the early stages in which gluten starts the whole process, but it has been shown that an inflammatory response precedes the antigen-specific T-cell response [21]. Some data suggest that non-MHC genes and/or non-gluten environmental factors are involved in the development of the disease [22]. In this regard, the group of Lunardi and Puccetti has identified a disease-relevant auto-antigen peptide sharing homology with the VP-7 protein of rotavirus. Autoantibodies directed against VP-7 protein are detected in patients with active CD and cross-react with a desmoglein peptide and TLR4 peptide. It has been proposed that rotavirus, through a mechanism of molecular mimicry, may induce production of antibodies against the VP-7 protein that are self-reactive antibodies and are pathogenically relevant for their ability to alter the intestinal barrier integrity and to activate monocytes by engaging TLR4 [23].

Some other studies have focused the attention on the effect of gluten on innate immune response. In fact, beside its ability to induce immune responses, wheat contains several toxic peptides, which are capable of inducing mucosal damage. These epitopes have sequence homology with prolamins of other cereals, such as barley, rye and potentially although in minor quantity in oat [24]. Several non-immunogenic gluten peptides that stimulate innate immune response but not the adaptive immune responses have been investigated. The most studied toxic peptide is the derived a-gliadin p31-43 that is part of the longer peptide 25-mer (Figure 1) and is resistant to digestive enzymes in the gut [15]. In 2003, Maiuri and colleagues demonstrated that the fragment p31-43 is able to activate innate immune response by macrophages, monocytes and dendritic cells of the *lamina propria*, without stimulating CD4+ T-cells [25]. Both in vitro and in vivo experiments demonstrated that the effects of p31-43 peptide are dependent on the adapter Myd88 and the type I IFNs and involve the MAPK and NF-κB signalling cascades [26,27].

Interestingly, the innate response to p31-43 peptide was detected only in HLA-DQ2 positive CD patients, since it was not found in HLA-DQ2 positive non-coeliac controls [25]. Even more intriguingly, anti-IL-15 neutralizing antibodies could inhibit all the events related to innate immune activation, thus showing that this cytokine plays a key role as mediator of intestinal mucosa damage induced by ingestion of gliadin. IL-15 is a regulatory cytokine secreted by cells of innate immunity, but not by T and B cells [28]. IL-15 supports the homeostasis between innate and adaptive immunity [29] and is involved in the expansion and migration of intraepithelial lymphocytes (IELs) [30]. Concerning IELs, IL-15 can lower the TCR threshold, making these cells able to kill targets that express IL-15 in a non-cognate yet TCR-dependent way [31]. Moreover, IL15 may also prime towards a Th1 response [32], directing the IFN-γ dominated gliadin T-cell activation and it has anti-apoptotic activities, protecting pathogenic CD4+ T-cells from death [33].

## 3. Role of MCs in Coeliac Disease

### 3.1. Intestinal MCs Biology

MCs are tissue-residents belonging to the innate arm of the immune system. In the gastrointestinal tract, they can be found in all layers but locate mainly in the *lamina propria* of the mucosa, where they comprise 1–5% of mononuclear cells, and less in the submucosa (about 1% of all cells) [34].

MCs originate from circulating precursors that enter the intestinal mucosa through the α4β7-MAdCAM-1 and α4β7-VCAM-1 interactions and under the influence of CXCR2 ligation [35,36]. Once in the intestinal mucosa, they acquire a unique mature phenotype, which consists in the expression on the cell membrane of the IgE-specific high affinity receptor and of the proteases-containing granules in the cytoplasm [37]. Historically, based on the protease content of their granules, MCs are classified as MCs containing tryptase but little or no chymase (MC_T_) and MCs containing tryptase, chymase and carboxypeptidase (MC_TC_). In the mucosa of the human small intestine, MC_T_ amount to 98% of all MCs while 77% of MCs resident in the sub-mucosa layer are MC_TC_ [38,39].

MCs are endowed with a wide pattern of membrane-bound receptors (FcεRI, FcγR, TLRs, protein G coupled receptors (GPCRs), chemokine and cytokine receptors) [40] and a great number of costimulatory molecules, including members of the B7 family (ICOSL, PD-L1, and PD-L2) and of the TNF/TNFR families (OX40L, CD153, Fas, 4-1BB, and glucocorticoid-induced TNFR), as shown in Table 1. This plethora of receptors empowers the MC with the ability to respond to a multitude of stimuli and to interact with different partners both from immune and non-immune cells populations [37]. Within the intestinal microenvironment, MCs are continuously exposed to different stimuli including antigen-bounded Ig, pathogens, exogenous (food antigens) as well as endogenous molecules such as neuropeptides, hormones, neurotransmitters and growth factors [37]. TLRs are fundamental in sensing foreign and pathogenic molecules whereas protein G coupled receptors are implicated with the recognition and binding of ligands of different origins ranging from antimicrobial peptides, neuropeptides, lipids, and adenosine or complement fragments. The expression of several members of both TLR [41] and GPCR families [42] confer to intestinal MCs the ability to sense every change in the local microenvironment composition and to rapidly mount a specific response.

The triggering of MC receptors causes the cell activation and degranulation—i.e., the quick empty of the granules content, followed by the neo-synthesis and secretion of many molecules with different biological activities that influence all stages of the immune cell response with both pro-inflammatory and immunosuppressive effect [43]. Furthermore, the same released mediators can also influence the biology of the neighbouring cells, namely the intestinal epithelial cells and cells of the nervous system, contributing to change of gut homeostasis (Table 2).

The rapid emptying of the cytoplasmic granules, which contain preformed immunomodulatory compounds such as histamine, serotonin, proteases, heparin, and TNF-α, has a strong effect on several functions of the human intestine. Through the activation of the four histamine receptor subtypes H1, H2; H3 and H4, histamine can induce both immunological response (H1-H4) as well as visceral nociception (H1) [44], gastric acid secretion [45] and increased intestinal motility [46].

Serotonin, (5-hydroxytryptamine, 5-HT) regulates many functions of the small intestine by virtue of its effect on neurones and intestinal cells and high levels of 5-HT are responsible of nausea, vomiting and diarrhoea [47]. Indeed, the augmented spontaneous release of 5-HT was found to correlate with increased MCs counts and with the severity of abdominal pain in inflammatory bowel disease [48]. Increased 5-HT content and enhanced 5-HT release from the upper small bowel have been identified in CD subjects and have been correlated with dyspeptic symptoms in untreated patients [49] leading to suppose a contribution of MC-derived 5-HT to the intestinal damage also in CD.

Tryptase activates a protease-activated receptor (PAR2) expressed on both the apical and basolateral side of the intestinal epithelial cells causing calcium mobilization, actin redistribution and Zonulin delocalisation [50,51] that consequently result in tight junctions’ disruption, cell apoptosis [52] and increased intestinal permeability [50,51,52].

Similarly, once released MC chymase cleaves several substrates that are important for tissue remodelling and extracellular matrix (ECM) degradation both directly and by activating ECM-degrading proteases, including matrix metalloproteases [53,54,55]. This ultimately results in tissue damage and loss of intestinal barrier integrity. Besides these pre-stored mediators with pronounced pro-inflammatory effect, MCs are, together with basophils, the unique cells of the immune system that store and rapidly release upon activation heparin. Apart from its anticoagulant activity, heparin plays an anti-inflammatory role in the intestinal mucosa as it attenuates the production of pro-inflammatory cytokines such as IL-6 and TNF-α [56], inhibits neutrophil recruitment and activation [57] showing a protective effect against intestinal mucosa damage.

Upon activation MCs release newly generated mediators, lipid derived (prostaglandin, leukotrienes and platelet activating factor) and neo-synthetized (cytokines and growth factors) molecules, that can have different effects on the gut homeostasis. In humans, PGD2 is the major prostaglandin while LTC4 and LTB4 are the most prevalent leukotrienes secreted by activated MCs [58]. Albeit a direct role of these mediators on intestinal permeability in human is still debated, in animals PGD2 and LTC4 were found to strongly induce intestinal secretion by direct actions on enteric neurons [59]. Among the pro-inflammatory cytokines that MCs release, TNF-α, IFN-γ and IL-6 were demonstrated to damage the intestinal epithelial barrier integrity by down-regulating the expression of occludin [60], claudin 2 [61], claudin 3 [62] respectively. The consequent reorganization of the protein architecture of the tight junctions alters the epithelial paracellular permeability.

Notably, the close proximity to sensory nerves in the intestinal mucosa permits the MCs to tightly communicate with the cells of the nervous system. These MCs-nerves interactions are bidirectional with neuronal activation that triggers the release of neuropeptides and neurotransmitters that thereby can bind MC receptors and induce the secretion of soluble mediators that ultimately activate neurons [63].Therefore, besides modulating innate and acquired immune response, intestinal MCs can influence peristalsis, vascular and epithelial permeability, ion secretion, nociception, angiogenesis and tissue repair, being crucially involved in the control of the gut homeostasis [64,65].

The ability of MCs to rapidly sense the modifications of the microenvironment and the ability to adapt their response to the specific received trigger, results in different phenotypes of the activated MCs described in different disorders of the intestine [66]. In the past, numerous studies have documented an accumulation of MCs in the intestinal mucosa of patients affected by many gastrointestinal diseases, including ulcerative colitis and inflammatory bowel disease (IBD), IBS, and in these contexts, MCs resulted to be fundamental mediators of the pathology-associated inflammation [67,68]. However, as stated by Theoharides and colleagues, the phenotype and the activation status of these MCs rather than their absolute numbers in the inflamed intestinal mucosa is relevant for the development and progression of the lesions [68].

In addition to these observations, it is worthy to note that MCs, in various tissues, express also receptors for sex hormones that regulate their functionality and tissue distribution both in physiological and pathological conditions [69]. Interestingly, sex hormones, estradiol, progesterone, and testosterone, were found to induce MC histamine release in a dose- and gender dependent way [70]. In this regard, a relationship between female hormones, MC-derived mediators and the development of allergic and gastrointestinal inflammatory diseases has been hypothesized [71,72]. In particular, oestrogen has been suggested to participate in pathogenesis of irritable bowel syndrome (IBS) [73]. Preliminary data on a newly described MC disorder, the mast cell activation syndrome, have shown that 89% of patients were female and that the most common symptoms affect the gastrointestinal tract, such as abdominal pain (94% of patients) and diarrhoea (67%). Interestingly, histologic and immunohistochemical analysis performed on biopsies of patients undergone to endoscopy showed no significant differences between patients and controls either in the number of intestinal MCs, or in the distribution of MCs. This suggests that there are differences in MC activity among different subjects, as well as between sex, with female MCs probably more prone to be activated. [74] Regarding CD, several studies have shown that 60% of patients are female and it has been reported that women have more severe disease than men. [75,76,77]. Thus, taken together these data suggest that MCs may sense and react to changes in the hormonal milieu, especially oestrogens. This physiologic behaviour may correspond in pathophysiology to a predisposition of women to develop gastrointestinal disorders and to have more severe gastrointestinal symptoms and disease burden than men.

### 3.2. Overview of Literature on MCs in CD

The first studies aiming at characterizing the role of intestinal mucosal MCs in CD date to 80s and produced conflicting results [68,69,70,71,72,73,74,75,76,77,78,79,80,81,82,83].

Earliest histological investigations of MCs within the intestinal mucosa (Table 3) documented a significantly decreased number of MCs in the *lamina propria* of CD patients as compared with controls. In 1979, Kumar and colleagues reported values to be higher than normal in untreated CD patients, remaining high after treatment with a gluten-free diet [78]. Similarly, in another report [79], MC counts were found to be increased in untreated CD, but returning to normal level after gluten withdrawal, while another study showed a positive correlation between increased values of infiltrating mucosal MCs and the heights of the villi in untreated CD, values remaining lower than normal after treatment [80]. Conversely, in the same years, both Dollberg [81] and Suranyi [82] demonstrated that significantly lower numbers of accumulating MCs were found in intestinal biopsies from untreated CD patients compared to healthy subjects, that return to the normal range in patients treated with a gluten-free diet. Moreover, in a study performed in 1986 by the group of Horvath, the histological analysis of the intestinal mucosa of 14 CD children showed a reduced number of MCs five hours after challenge with gluten [83].

These discrepancies were probably caused by errors in the analysis of intestinal biopsy specimens due to inappropriate employed methodology. Indeed, these studies were mainly retrospective analysis of tissues, which had been processed for diagnostic purposes, and probably they were not handled properly for the identification of MCs. Thus, these earlier conflicting observations were attributable to different fixative-associated blocking agents and staining procedures, which impair the adequate histochemical visualization of the MC granules.

However, in 1989, a first functional study documented a potential active role of MCs in the mechanism of gluten-induced jejunal damage [84]. In the paper by Lavö, the authors observed that in vitro gluten perfusion of small intestinal biopsies from CD patients induced a two-fold increase of histamine secretion within 40 minutes, but did not influence the secretion rates of histamine in biopsies from healthy controls [84].

Related experiments aimed to investigate the primary response of immune cells and MCs to local gluten challenge were performed by other groups and gave similar results. In their studies on rectal mucosa, Loft and colleagues demonstrated a strong reduction in the number of toluidine-blue stained MCs in rectal biopsies of CD patients exposed to gluten for 24 hours and ascribed this reduction in granulated MCs to the empty of granules as consequence of the degranulation response to gliadin [85]. Similarly, a decrease in degranulated MCs was observed in the oral mucosa of CD patients after gluten challenge. In fact, despite the comparable numbers of MCs in the *lamina propria* of the oral mucosa of CD patients and controls before gliadin challenge, after gliadin injection into the oral submucosa a significant reduction in the total numbers of MCs was found in CD patients but not in controls [86].

These were the first evidences of a direct response of MCs to gluten challenge. Nevertheless, the difficulties in the identification and isolation of intestinal MCs have precluded inferring clues about the role of MCs in the CD pathogenesis and pathophysiology for long time (Table 1). Indeed, only in 2017 new studies on the role of MCs in CD were published.

**Figure 2:**
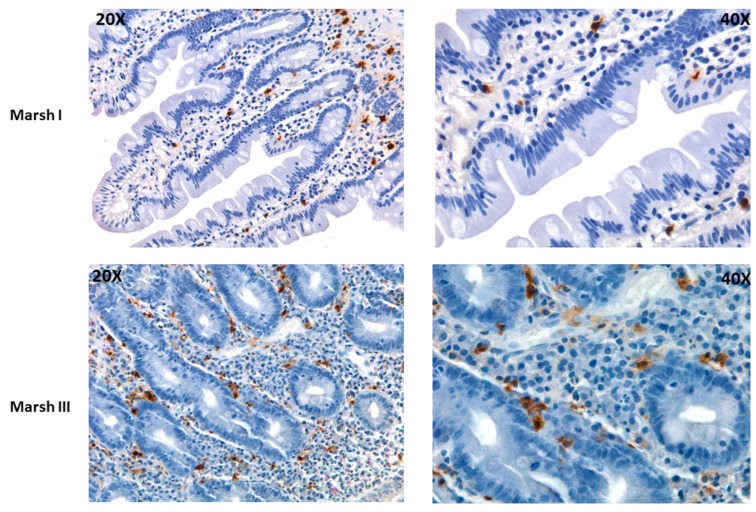
Mast cell accumulation in Coeliac Disease patients increases with the extent of histological damage. Immunohistochemical staining for tryptase in duodenal specimens of two patients with Marsh 1 and Marsh 3 scores. Staining is shown at 20× (left side) and 40× magnifications (right side). Images were kindly provided by prof. Claudio Tripodo and Dr. Beatrice Belmonte.

In a recent work of Losurdo and colleagues, 20 subjects with non-coeliac gluten sensitivity (NCGS) and 16 CD patients (classified as Marsh 1 and Marsh 2) were enrolled to investigate the specific expression of markers of adaptive and innate immunity activation in the course of the disease. The authors specifically identified intestinal MCs by CD117 staining (i.e., the receptor for SCF that is constitutively expressed by mature MCs) and demonstrated that MCs accumulate in the intestinal mucosa of both NCGS and CD patients [87]. In the same year, by analysing and scoring the intestinal biopsies of CD patients for immune cells infiltration (including B, T-cells as well as macrophages and MCs) according to Marsh classification, the histological damage progression in CD has been associated with the density of infiltrating MCs (Figure 2) and their ability to bind the non-immunogenic gliadin p31-43 peptide and to release inflammatory mediators in response to p31-43 [88].

### 3.3. MC as a New Player in CD

In the study carried out in 2017 on human MC lines and primary cultures of intestinal MCs, we proved a direct role of these cells in onset of the immune response to gliadin, and in keeping disease progression [88]. We observed that both LAD2 human MCs and intestinal mucosal MCs selectively react to the treatment with the non-immunogenic p31-43 fragment of the gliadin but not to other gluten proteins, or to gliadin immunogenic peptides. The p31-43 fragment is the only one capable of inducing the release of histamine and specific pro-inflammatory cytokines through the generation of reactive oxygen species and the activation of the transcription factor NF-κB. By using primary cultures of MCs obtained from mice genetically deficient of the Myd-88 adaptive molecule, we also showed that p31-43 requires the TLR pathway to induce MC activation [88].

Moreover, different in vitro responsiveness to p31-43 peptide stimulation was observed: intestinal MCs isolated from healthy subjects responded less (in term of histamine release) than MCs isolated from CD subjects, and the magnitude of MC response is directly associated to the severity of intestinal lesions from which they stem. Indeed, MCs isolated from the intestinal mucosa of CD patients classified as Marsh 3 release more histamine in response to p31-43 peptide than MCs isolated from Marsh 1 patients [88].

Among all the cells infiltrating the intestinal mucosa of CD subjects, we have also shown that MCs are the only cells that accumulate proportionally to the worsening of the extent of tissue damage (Figure 2). Moreover, such numerical increase is accompanied by a phenotypic change of intestinal MCs, which progressively become a cellular source of TNF-, IL-6 and IL-17 [88].

**Figure 3:**
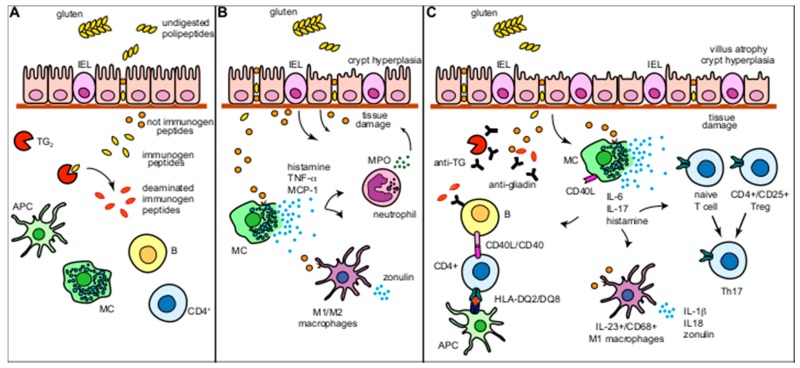
Schematic representation of mast cell’s (MC) role in the pathophysiology of the coeliac disease (CD). (**A**) Under basal condition, MCs localize mainly in the *lamina propria* of the intestinal mucosa. Undigested gluten peptides cross the epithelial barrier and reach the *lamina propria* where they are deaminated locally by the tissue transglutaminase 2 (TG2). Immunogenic deaminated peptides can bind the HLA-DQ2 or HLA-DQ8 molecules and can be presented to T-helper cells resulting in adaptive immune response activation. Non-immunogenic peptides can bind and activate the response of innate immune cells. (**B**) Tissue resident MCs can directly react to non-immunogenic toxic p31-43 fragment of gliadin by releasing preformed and newly synthetized mediators. These mediators are able to recruit and activate local macrophage and neutrophils contributing with MC to generate the tissue damage in the early phase of the disease (Marsh 1). (**C**) In the intestinal lesions of CD patients characterized by high Marsh grades (Marsh 2 and 3), MCs become a significant source of pro-inflammatory cytokines (IL-6 and IL-17). By the release of IL-6 and by the expression of CD40L, MC sustain B cell in the production of IgA, while MC-derived IL-6 and IL-17 promote the skew of local Treg cells into Th17. The cytokine milieu created by MCs also favors the polarization of the microenvironment toward a M1 phenotype. APC, antigen presenting cell; MPO, myeloperoxidase; TG2, transglutaminase 2.

Thus, if the release by MCs of histamine and inflammatory mediators following the trigger with gliadin p31-43 peptide could be accountable for the increase of leukocytes and of PMNs in the early stage of the disease [89] (Figure 3B), the subsequent increase in the production of pro-inflammatory cytokine (IL-6 and IL-17 mainly) by intestinal MCs will be accountable for T- and B-cell activation (Figure 3C). Indeed, the cytokine milieu that MCs generate in response to gliadin activation contributes to intraepithelial T-cell activation and Th17 cell expansion and supports the skewing towards M1 phenotype of macrophages that will sustain T-cell response via presentation of immunogenic gluten peptides. Moreover, gliadin-activated MCs can also influence—in a T-independent way—the proliferation and differentiation of B cells into IgA-producing plasma cells owing to the constitutive expression of CD40L and the secretion of IL-6 [90] in response to p31-43 peptide challenge.

## 4. Conclusions and Future Perspectives

Even though finding a moderate correlation between a cytokine and the worsening of the disease is not possible, it is worth to note that during CD progression MCs increase in number and contribute to define a pro-inflammatory microenvironment. Considering that MCs are at the forefront in the interaction with the environment due to their privileged position within the mucosal tissue, the ability of MCs to respond to gliadin peptides accounts *firstly* for a direct role of MCs in the onset of CD. The ability of MCs, as monocytes and macrophages, to directly react against gluten challenge strongly supports the contribution of the innate immune system in initiating the immune response to gluten. *Secondly*, the observed changes in type and amounts of soluble mediators released by MCs during the progression of the disease indicate that the behaviour of MCs is not fixed and varies in response to microenvironment modifications. The association of different phenotypes of MCs with different histological degrees of intestinal lesions suggests that these modifications can be ascribed to the MCs change in cytokine profile or activity. In other words, the characterization of the phenotype of intestinal MCs could represent a “snapshot” of the status of the CD progression.

To date MCs cannot be taken as a truly marker of CD and further work is needed to better understand the role of these type of cells in the different setting of CD (i.e., refractory CD, in the same patient before and after gluten free diet, etc.). However, the presence of MCs from the beginning of the disease as well as their modifications over time are in favor of a plastic and active role of these cells that could be modulated by external triggers. Medications able to control MC activity are not jet available but the evidence that MCs can be triggered by diverse stimuli should direct researchers toward the identification of specific molecules to tune MCs.

In conclusion, it can be assumed that MCs represent one of the main players of the intestinal damage in the onset of CD. Hence, the pathogenesis of CD disease should be revised and the contribution of MCs in the onset and progression of the disease should be considered in the planning of new therapeutic approaches.

## Figures and Tables

**Table 1:** Mast Cell (MC) receptors.

Receptor Family	Members	Ligands
**FcR**		
FcεR	FcεRI	IgE
FcγR	FcγRI, FcγRII, FcγRIII	IgG
**Toll like receptors**	TLR1, TLR2, TLR3, TLR4, TLR5, TLR6, TLR7, TLR8, TLR9	Microbial PAMPs
**G protein coupled receptor**	MRGPRX2	Antimicrobial host defense peptides, neuropeptides, major basic protein, substance P, vaso intestinal peptide
**Adhesion molecules**	ICAM-1, VCAM, VLA4, Siglec6, Siglec7, Siglec8, SynCAM, N-cadherin, α2β1, α3β1, α4β1, α5β1, αVβ3	LFA-1, VLA-4, α4β1, sialic acid, fibronectin, collagen
**Co-stimulatory molecules**		
TNF/TNFR family member	CD40L, OX40L, 4-1BB, GITR, CD153, Fas, TRAIL-R	CD40, OX40, 4-1BBL, GITR ligand, CD30, FasL, TRAIL
B7 family members	CD28, ICOSL, PD-L1, PD-L2	CD80, CD86, ICOS, PD1, PD2
Nothch family members	Notch1, Notch2	Jag1, Jag2, Delta1, Delta4

TNF, tumor necrosis factor; TNFR, tumor necrosis factor receptor.

**Table 2:** Major MC-derived mediators and their effect on gastrointestinal (GI) mucosa.

Class	Mediator	Effect on GI	Ref.
**Pre-formed**			
*Biogenic amines*	Histamine	Neuron excitation Increased Cl^−^ secretion Increased intestinal motility	[44] [45] [46]
	5-hydroxytryptamine	Neuron excitation	[47,48,49]
*Proteases*	Tryptase	TJ disruption Increased intestinal permeability Epithelial cell apoptosis	[50] [50,51] [52]
	Chymase	ECM degradation Increased intestinal permeability	[53,54,55] [55]
*Proteoglicans*	Heparin	Inhibition of cytokine and chemokines production	[56,57,58]
**Newly-synthesized**			
*Lipid-derived*	PGD2	Increased electrolytic secretion	[59]
	LTC4, LTD4	Increased electrolytic secretion	[59,60]
*Cytokines*	IL-1, IL-6, IL-17, TNF-α	TJ disruption	[61,62,63]
*Neuropeptides*	Vasoactive intestinal peptide, NGF, SP	Neuron excitation	[64,65,66]

ECM, extracellular matrix; TJ, tight junctions.

**Table 3:** MC implication in coeliac disease (CD).

Subjects	MC or MC Product Identification	Observation/Effect	Reference
20 CD patients untreated and on gluten-free diet	Astra blu staining	MC numbers are higher than normal in intestinal mucosa of untreated CD patients and remain high after gluten-free diet	[70]
25 pediatric CD patients 10 pediatric CD patients on gluten-free diet	Astra blu staining	Lower number of (granulated) MCs in intestinal mucosa of untreated CD patients compared to patients on gluten-free diet Decrease in number of (granulated) MCs in patients on gluten-free diet after receiving gluten	[73]
19 CD patients 11 CD patients on gluten-free diet	Astra blu/safranin pH 0.3 staining	Increase number of MCs in intestinal mucosa of untreated CD patients returning to normal range after gluten-free diet	[71]
47 pediatric CD patients 17 pediatric CD patients on gluten-free diet	Iron diamine staining	Positive correlation between number of MCs and villous height Increased number of (granulated) MCs in patients under diet regimen	[72]
20 pediatric CD patients untreated and on gluten-free diet	Toluidine blu staining	During the untreated phase of the disease the MC number in the intestinal mucosa is depressed. On a gluten-free diet the number of MCs rises	[74]
14 children with treated CD disease	Toluidine blu staining	Reduced number of intestinal MCs 5h after single challenge with gluten	[75]
10 adult CD patients	Histamine release detection	Gliadin perfusion of closed jejunal segment induces a twofold histamine secretion in CD patients compared to control	[76]
10 adult CD patients on gluten-free diet	Toluidine blu staining	Rapid reduction (1h) of MC numbers in the rectal mucosa of CD patiets after instillation of gluten solution into the rectum	[77]
37 adult CD patients on gluten-free diet	Toluidine blu staining	Reduced numbers of (granulated) MCs in the oral submucosa of CD patients after injection of gliadin solution into the buccal submucosa	[78]
20 Non-coeliac gluten sensitivity (NCGS) patients 16 CD patients (Marsh 1–2)	CD117 staining	CD117+ cells are higher in jeunal biopsies of NCGS and CD patients compared to control	[79]
10 Marsh1 CD patients 9 Marsh2 CD patients 9 Marsh3 CD patients	Tryptase staining	Increased numbers of trptase+ cells in intestinal mucosa of Marsh 2 and Marsh 3 compare to Marsh 1 CD patients and controls The disease worsening associates with the acquisition of a pro-inflammatory phenotype of MCs	[80]

## References

[1] Ludvigsson J.F., Leffler D.A., Bai J.C., Biagi F., Fasano A., Green P.H., Hadjivassiliou M., Kaukinen K., Kelly C.P., Leonard J.N. (2013). The Oslo definitions for coeliac disease and related terms. Gut.

[2] Mustalahti K., Catassi C., Reunanen A., Fabiani E., Heier M., McMillan S., Murray L., Metzger M.H., Gasparin M., Bravi E. (2010). The prevalence of coeliac disease in Europe: Results of a centralized, international mass screening project. Ann. Med..

[3] Bibbò S., Pes G.M., Usai-Satta P., Salis R., Soro S., Quarta Colosso B.M., Dore M.P. (2017). Chronic autoimmune disorders are increased in coeliac disease: A case-control study. Medicine.

[4] Oberhuber G., Granditsch G., Vogelsang H. (1999). The histopathology of coeliac disease: Time for a standardized report scheme for pathologists. Eur. J. Gastroenterol. Hepatol..

[5] Dieterich W., Ehnis T., Bauer M., Donner P., Volta U., Riecken E.O., Schuppan D. (1997). Identification of tissue transglutaminase as the autoantigen of coeliac disease. Nat. Med..

[6] Van Elburg R.M., Uil J.J., Mulder C.J., Heymans H.S. (1993). Intestinal permeability in patients with coeliac disease and relatives of patients with coeliac disease. Gut.

[7] Matysiak-Budnik T., Candalh C., Dugave C., Namane A., Cellier C., Cerf-Bensussan N., Heyman M. (2003). Alterations of the intestinal transport and processing of gliadin peptides in coeliac disease. Gastroenterology.

[8] Molberg Ø., Solheim Flaete N., Jensen T., Lundin K.E., Arentz-Hansen H., Anderson O.D., Kjersti Uhlen A., Sollid L.M. (2003). Intestinal esponses to high-molecular-weight glutenins in coeliac disease. Gastroenterology.

[9] Shan L., Molberg Ø., Parrot I., Hausch F., Filiz F., Gray G.M., Sollid L.M., Khosla C. (2002). Structural basis for gluten intolerance in coeliac sprue. Science.

[10] Molberg O., Mcadam S.N., Körner R., Quarsten H., Kristiansen C., Madsen L., Fugger L., Scott H., Norén O., Roepstorff P. (1998). Tissue transglutaminase selectively modifies gliadin peptides that are recognized by gut-derived T cells in coeliac disease. Nat. Med..

[11] Nilsen E.M., Lundin K.E., Krajci P., Scott H., Sollid L.M., Brandtzaeg P. (1995). Gluten specific, HLA-DQ restricted T cells from coeliac mucosa produce cytokines with Th1 or Th0 profile dominated by interferon gamma. Gut.

[12] Nilsen E.M., Jahnsen F.L., Lundin K.E., Johansen F.E., Fausa O., Sollid L.M., Jahnsen J., Scott H., Brandtzaeg P. (1998). Gluten induces an intestinal cytokine response strongly dominated by interferon gamma in patients with coeliac disease. Gastroenterology.

[13] Bodd M., Ráki M., Tollefsen S., Fallang L.E., Bergseng E., Lundin K.E., Sollid L.M. (2010). HLA-DQ2-restricted gluten-reactive T cells produce IL-21 but not IL-17 or IL-22. Mucosal. Immunol..

[14] Cicerone C., Nenna R., Pontone S. (2015). Th17, intestinal microbiota and the abnormal immune response in the pathogenesis of coeliac disease. Gastroenterol. Hepatol. Bed. Bench..

[15] Mamone G., Ferranti P., Rossi M., Roepstorff P., Fierro O., Malorni A., Addeo F. (2007). Identification of a peptide from α-gliadin resistant to digestive enzymes: Implications for coeliac disease. J. Chromatogr. B Analyt. Technol. Biomed. Life Sci..

[16] Qiao S.W., Bergseng E., Molberg Ø., Xia J., Fleckenstein B., Khosla C., Sollid L.M. (2004). Antigen presentation to coeliac lesion-derived T cells of a 33-mer gliadin peptide naturally formed by gastrointestinal digestion. J. Immunol..

[17] Green P.H., Jabri B. (2003). Coeliac disease. Lancet.

[18] Jabri B., Abadie V. (2015). IL-15 functions as a danger signal to regulate tissue-resident T cells and tissue destruction. Nat. Rev. Immunol..

[19] Gianfrani C., Troncone R., Mugione P., Cosentini E., De Pascale M., Faruolo C., Senger S., Terrazzano G., Southwood S., Auricchio S. (2003). Coeliac disease association with CD8^+^ T cell responses: Identification of a novel gliadin-derived HLA-A2-restricted epitope. J. Immunol..

[20] Mazzarella G., Stefanile R., Camarca A., Giliberti P., Cosentini E., Marano C., Iaquinto G., Giardullo N., Auricchio S., Sette A. (2008). Gliadin activates HLA class I-restricted CD8^+^ T cells in coeliac disease intestinal mucosa and induces the enterocyte apoptosis. Gastroenterology.

[21] Maiuri L., Picarelli A., Boirivant M., Coletta S., Mazzilli M.C., De Vincenzi M., Londei M., Auricchio S. (1996). Definition of the initial immunologic modifications upon in vitro gliadin challenge in the small intestine of coeliac patients. Gastroenterology.

[22] Abadie V., Sollid L.M., Barreiro L.B., Jabri B. (2011). Integration of genetic and immunological insights into a model of coeliac disease pathogenesis. Annu. Rev. Immunol..

[23] Zanoni G., Navone R., Lunardi C., Tridente G., Bason C., Sivori S., Beri R., Dolcino M., Valletta E., Corrocher R. (2006). In coeliac disease, a subset of autoantibodies against transglutaminase binds toll-like receptor 4 and induces activation of monocytes. PLoS Med..

[24] Balakireva A.V., Zamyatnin A.A. (2016). Properties of Gluten Intolerance: Gluten Structure, Evolution, Pathogenicity and Detoxification Capabilities. Nutrients.

[25] Maiuri L., Ciacci C., Ricciardelli I., Vacca L., Raia V., Auricchio S., Picard J., Osman M., Quaratino S., Londei M. (2003). Association between innate response to gliadin and activation of pathogenic T-cells in coeliac disease. Lancet.

[26] Palová-Jelínková L., Dáňová K., Drašarová H., Dvořák M., Funda D.P., Fundová P., Kotrbová-Kozak A., Černá M., Kamanová J., Martin S.F. (2013). Pepsin digest of wheat gliadin fraction increases production of IL-1β via TLR4/MyD88/TRIF/MAPK/NF-κB signaling pathway and an NLRP3 inflammasome activation. PLoS ONE.

[27] Araya R.E., Gomez Castro M.F., Carasi P., McCarville J.L., Jury J., Mowat A.M., Verdu E.F., Chirdo F.G. (2016). Mechanisms of innate immune activation by gluten peptide p31-43 in mice. Am. J. Physiol. Gastrointest. Liver Physiol..

[28] Patidar M., Yadav N., Dalai S.K. (2016). Interleukin 15: A key cytokine for immunotherapy. Cytokine Growth Factor Rev..

[29] Tagaya Y., Bamford R.N., DeFilippis A.P., Waldmann T.A. (1996). IL-15: A pleiotropic cytokine with diverse receptor/signaling pathways whose expression is controlled at multiple levels. Immunity.

[30] Ebert E.C. (1998). Interleukin 15 is a potent stimulant of intraepithelial lymphocytes. Gastroenterology.

[31] Liu R.B., Engels B., Schreiber K., Ciszewski C., Schietinger A., Schreiber H., Jabri B. (2013). IL-15 in tumor microenvironment causes rejection of large established tumors by T cells in a noncognate T-cell receptor-dependent manner. Proc. Natl. Acad. Sci. USA.

[32] Fehniger T.A., Caligiuri M.A. (2001). Interleukin 15: Biology and relevance to human disease. Blood.

[33] Londei M., Quaratino S., Maiuri L. (2003). Coeliac disease: A model autoimmune disease with gene therapy applications. Gene Ther..

[34] Bischoff S.C. (2009). Physiological and pathophysiological functions of intestinal MCs. Semin. Immunopathol..

[35] Gurish M.F., Tao H., Abonia J.P., Arya A., Friend D.S., Parker C.M., Austen K.F. (2001). Intestinal MCs progenitors require CD49dβ7 (α4β7 integrin) for tissue-specific homing. J. Exp. Med..

[36] Abonia J.P., Austen K.F., Rollins B.J., Joshi S.K., Flavell R.A., Kuziel W.A., Koni P.A., Gurish M.F. (2005). Constitutive homing of MCs progenitors to the intestine depends on autologous expression of the chemokine receptor CXCR2. Blood.

[37] Frossi B., Mion F., Tripodo C., Colombo M.P., Pucillo C.E. (2017). Rheostatic Functions of MCs in the Control of Innate and Adaptive Immune Responses. Trends Immunol..

[38] Weidner N., Austen K.F. (1993). Heterogeneity of mast cells at multiple body sites: Fluorescent determination of avidin binding and immunofluorescent determination of chymase, tryptase, and carboxypeptidase content. Pathol. Res. Pract..

[39] Irani A.M., Bradford T.R., Kepley C.L., Schechter N.M., Schwartz L.B. (1989). Detection of MCT and MCTC types of human mast cells by immunohistochemistry using new monoclonal anti-tryptase and anti-chymase antibodies. J. Histochem Cytochem..

[40] Redegeld F.A., Yu Y., Kumari S., Charles N., Blank U. (2018). Non-IgE mediated mast cell activation. Immunol. Rev..

[41] Sandig H., Bulfone-Pauss S. (2012). TLR signaling in mast cell: Common and unique features. Front. Immunol..

[42] Subramanian H., Gupta K., Hydar A. (2016). Roles of MAS-related G protein coupled receptor-X2 (MRGPRX2) on mast cell-mediated host defense, pseudoallergic drug reactions and chronic inflammatory diseases. J. Allergy Clin Immunol..

[43] Gri G., Frossi B., D’Inca F., Danelli L., Betto E., Mion F., Sibilano R., Pucillo C. (2012). Mast cell: An emerging partner in immune interaction. Front. Immunol..

[44] Deiteren A., De Man J.G., Pelckmans P.A., De Winter B.Y. (1995). Histamine H4 receptors in the gastrointestinal tract. Br. J. Pharmacol..

[45] Homaidan F.R., Tripodi J., Zhao L., Burakoff R. (1997). Regulation of ion transport by histamine in mouse cecum. Eur. J. Pharmacol..

[46] Barbara G., Wang B., Stanghellini V., de Giorgio R., Cremon C., Di Nardo G., Trevisani M., Campi B., Geppetti P., Tonini M. (2007). Mast cell-dependent excitation of visceral-nociceptive sensory neurons in irritable bowel syndrome. Gastroenterology.

[47] Mawe G.M., Hoffman J.M. (2013). Serotonin signalling in the gut--functions, dysfunctions and therapeutic targets. Nat. Rev. Gastroenterol. Hepatol..

[48] Cremon C., Carini G., Wang B., Vasina V., Cogliandro R.F., De Giorgio R., Stanghellini V., Grundy D., Tonini M., De Ponti F. (2011). Intestinal serotonin release, sensory neuron activation, and abdominal pain in irritable bowel syndrome. Am. J. Gastroenterol..

[49] Coleman N.S., Foley S., Dunlop S.P., Wheatcroft J., Blackshaw E., Perkins A.C., Singh G., Marsden C.A., Holmes G.K., Spiller R.C. (2006). Abnormalities of serotonin metabolism and their relation to symptoms in untreated celiac disease. Clin. Gastroenterol. Hepatol..

[50] Wilcz-Villega E.M., McClean S., O’Sullivan M.A. (2013). Mast cell tryptase reduces junctional adhesion molecule-A (JAM-A) expression in intestinal epithelial cells: Implications for the mechanisms of barrier dysfunction in irritable bowel syndrome. Am. J. Gastroenterol..

[51] Jacob C., Yang P.C., Darmoul D., Amadesi S., Saito T., Cottrell G.S., Coelho A.M., Singh P., Grady E.F., Perdue M. (2005). Mast cell tryptase controls paracellular permeability of the intestine. Role of protease-activated receptor 2 and β-arrestins. J. Biol. Chem..

[52] Li S., Guan J., Ge M., Huang P., Lin Y., Gan X. (2015). Intestinal mucosal injury induced by tryptase-activated protease-activated receptor 2 requires β-arrestin-2 in vitro. Mol. Med. Rep..

[53] Tchougounova E., Lundequist A., Fajardo I., Winberg J.-O., Åbrink M., Pejler G. (2005). A key role for mast cell chymase in the activation of pro-matrix metalloprotease-9 and pro-matrix metalloprotease-2. J. Biol. Chem..

[54] Groschwitz K.R., Wu D., Osterfeld H., Ahrens R., Hogan S.P. (2013). Chymase-mediated intestinal epithelial permeability is regulated by a protease-activating receptor/matrix metalloproteinase-2-dependent mechanism. Am. J. Physiol. Gastrointest. Liver Physiol..

[55] Fu Z., Thorpe M., Hellman L. (2015). rMCP-2, the Major Rat Mucosal Mast Cell Protease, an Analysis of Its Extended Cleavage Specificity and Its Potential Role in Regulating Intestinal Permeability by the Cleavage of Cell Adhesion and Junction Proteins. PLoS ONE.

[56] Papa A., Danese S., Gasbarrini A., Gasbarrini G. (2000). Review article: Potential therapeutic applications and mechanisms of action of heparin in inflammatory bowel disease. Aliment Pharmacol. Ther..

[57] Salas A., Sans M., Soriano A., Reverter J.C., Anderson D.C., Piqué J.M., Panés J. (2000). Heparin attenuates TNF-α induced inflammatory response through a CD11b dependent mechanism. Gut.

[58] Boyce J.A. (2005). Eicosanoid mediators of mast cells: Receptors, regulation of synthesis, and pathobiologic implications. Chem. Immunol. Allergy.

[59] Sharkey K.A., Kroese A.B. (2001). Consequences of intestinal inflammation on the enteric nervous system: Neuronal activation induced by inflammatory mediators. Anat. Rec..

[60] Mankertz J., Tavalali S., Schmitz H., Mankertz A., Riecken E.O., Fromm M., Schulzke J.D. (2000). Expression from the human occludin promoter is affected by tumor necrosis factor α and interferon gamma. J. Cell Sci..

[61] Prasad S., Mingrino R., Kaukinen K., Hayes K.L., Powell R.M., MacDonald T.T., Collins J.E. (2005). Inflammatory processes have differential effects on claudins 2, 3 and 4 in colonic epithelial cells. Lab. Investig..

[62] Al-Sadi R., Ye D., Boivin M., Guo S., Hashimi M., Ereifej L., Ma T.Y. (2014). Interleukin-6 modulation of intestinal epithelial tight junction permeability is mediated by JNK pathway activation of claudin-2 gene. PLoS ONE.

[63] Van Nassauw D., Adriaensen J.P. (2007). Timmermans The bidirectional communication between neurons and mast cells within the gastrointestinal tract. Auton. Neurosci..

[64] Buhner S., Schemann M. (2012). Mast cell-nerve axis with a focus on the human gut. Biochim. Biophys. Acta.

[65] van Diest S.A., Stanisor O.I., Boeckxstaens G.E., de Jonge W.J., van den Wijngaard R.M. (2012). Relevance of MCs-nerve interactions in intestinal nociception. Biochim. Biophys. Acta.

[66] Frossi B., Mion F., Sibilano R., Danelli L., Pucillo C.E.M. (2018). Is it time for a new classification of mast cells? What do we know about mast cell heterogeneity?. Immunol. Rev..

[67] Wouters M.M., Vicario M., Santos J. (2016). The role of MCs in functional GI disorders. Gut.

[68] Theoharides T.C. (2014). MCs in Irritable Bowel Syndrome and Ulcerative Colitis: Function Not Numbers Is What Makes All the Difference. Dig. Dis. Sci..

[69] Kim M., Chae H., Shin T., Kim H., Kim H. (2001). Estrogen regulates cytokine release in human mast cells. Immunopharmacol. Immunotoxicol..

[70] Muñoz-Cruz S., Mendoza-Rodríguez Y., Nava-Castro K.E., Yepez-Mulia L., Morales-Montor J. (2015). Gender-related effects of sex steroids on histamine release and FcεRI expression in rat peritoneal mast cells. J. Immunol. Res..

[71] Zierau O., Zenclussen A.C., Jensen F. (2012). Role of female sex hormones, estradiol and progesterone, in mast cell behaviour. Front. Immunol..

[72] Chen W., Mempel M., Schober W., Behrendt H., Ring J. (2008). Gender difference, sex hormones, and immediate type hypersensitivity reactions. Allergy.

[73] Zielińska M., Fichna J., Bashashati M., Habibi S., Sibaev A., Timmermans J.P., Storr M. (2017). G protein-coupled estrogen receptor and estrogen receptor ligands regulate colonic motility and visceral pain. Neurogastroenterol. Motil..

[74] Hamilton M., Hornick J.L., Akin C., Castells M.C., Greenberger N.J. (2011). Mast cell activation syndrome: A newly recognized disorder with systemic clinical manifestations. J. Allergy Clin. Immunol..

[75] Jansson-Knodell C.L., Hujoel I.A., West C.P., Taneja V., Prokop L.J., Rubio-Tapia A., Murray J.A. (2018). Sex Difference in Celiac Disease in Undiagnosed Populations: A Systematic Review and Meta-analysis. Clin. Gastroenterol. Hepatol..

[76] Ciacci C., Cirillo M., Sollazzo R., Savino G., Sabbatini F., Mazzacca G. (1995). Gender and clinical presentation in adult celiac disease. Scand. J. Gastroenterol..

[77] Ludvigsson J.F., Montgomery S.M., Ekbom A., Brandt L., Granath F. (2009). Small-intestinal histopathology and mortality risk in celiac disease. JAMA.

[78] Kumar P.O., Donoghue D.P., Stenson K., Dawson A.M. (1979). Reintroduction of gluten in children with treated coeliac disease. Gut.

[79] Strobel S., Busuttil A., Ferguson A. (1983). Human intestinal mucosal MCs: Expanded population in untreated coeliac disease. Gut.

[80] Kosnai I., Kuitunen P., Savilahti E., Sipponen P. (1984). Mast cells and eosinophils in the jejunal mucosa of patients with intestinal cow’s milk allergy and coeliac disease of childhood. J. Pediatr. Gastroenterol. Nutr..

[81] Dollberg L., Gurevitz M., Freier S. (1980). Gastrointestinal MCs in health, and in coeliac disease and other conditions. Arch. Dis. Childh..

[82] Suranyi Y., Freier S., Faber J., Dollberg L. (1986). Intestinal mast cells in different stages of coeliac disease. Isr. J. Med. Sci..

[83] Horváth K., Simon K., Horn G., Bodánszky H. (1986). Mast cell degranulation after a single dose of gliadin in the jejunum of patients with coeliac disease. Acta Paediatr. Hung..

[84] Lavö B., Knutson L., Lööf L., Odlind B., Venge P., Hällgren R. (1989). Challenge with gliadin induces eosinophil and MCs activation in the jejunum of patients with coeliac disease. Am. J. Med..

[85] Loft D.E., Marsh M.N., Sandle G.I., Crowe P.T., Garner V., Gordon D., Baker R. (1989). Studies of intestinal lymphoid tissue. XII. Epithelial lymphocyte and mucosal responses to rectal gluten challenge in celiac sprue. Gastroenterology.

[86] Lähteenoja H., Mäki M., Viander M., Räihä I., Vilja P., Rantala I., Toivanen A., Syrjänen S. (2000). Local challenge on oral mucosa with an α-gliadin related synthetic peptide in patients with celiac disease. Am. J. Gastroenterol..

[87] Losurdo G., Piscitelli D., Pezzuto F., Fortarezza F., Covelli C., Marra A., Iannone A., Amoruso A., Principi M., Ierardi E. (2017). T Helper Lymphocyte and Mast Cell Immunohistochemical Pattern in Non-coeliac Gluten Sensitivity. Gastroenterol. Res. Pract..

[88] Frossi B., Tripodo C., Guarnotta C., Carroccio A., De Carli M., De Carli S., Marino M., Calabrò A., Pucillo C.E. (2017). MCs are associated with the onset and progression of coeliac disease. J. Allergy Clin. Immunol..

[89] Enoksson M., Moller-Westerberg C., Wicher G., Fallon P.G., Forsberg-Nilsson K., Lunderius-Andersson C., Nilsson G. (2013). Intraperitoneal influx of neutrophils in response to IL-33 is mast cell-dependent. Blood.

[90] Merluzzi S., Frossi B., Gri G., Parusso S., Tripodo C., Pucillo C. (2010). Mast cells enhance proliferation of B lymphocytes and drive their differentiation toward IgA-secreting plasma cells. Blood.

